# Role of *HLA*, *KIR*, *MICA*, and Cytokines Genes in Leprosy

**DOI:** 10.1155/2013/989837

**Published:** 2013-07-08

**Authors:** Luciana Ribeiro Jarduli, Ana Maria Sell, Pâmela Guimarães Reis, Emília Ângela Sippert, Christiane Maria Ayo, Priscila Saamara Mazini, Hugo Vicentin Alves, Jorge Juarez Vieira Teixeira, Jeane Eliete Laguila Visentainer

**Affiliations:** ^1^Program of Biosciences Applied to Pharmacy, Department of Clinical Analysis and Biomedicine, Maringa State University, Avenida Colombo 5790, 87020-900 Maringá, PR, Brazil; ^2^Basic Health Sciences Department, Maringa State University, Avenida Colombo 5790, 87020-900 Maringá, PR, Brazil

## Abstract

Many genes including *HLA*, *KIR*, and *MICA* genes, as well as polymorphisms in cytokines have been investigated for their role in infectious disease. *HLA* alleles may influence not only susceptibility or resistance to leprosy, but also the course of the disease. Some combinations of *HLA* and *KIR* may result in negative as well as positive interactions between NK cells and infected host cells with *M. leprae*, resulting in activation or inhibition of NK cells and, consequently, in death of bacillus. In addition, studies have demonstrated the influence of *MICA* genes in the pathogenesis of leprosy. Specifically, they may play a role in the interaction between NK cells and infected cells. Finally, pro- and anti-inflammatory cytokines have been influencing the clinical course of leprosy. Data from a wide variety of sources support the existence of genetic factors influencing the leprosy pathogenesis. These sources include twin studies, segregation analyses, family-based linkage and association studies, candidate gene association studies, and, most recently, genome-wide association studies (GWAS). The purpose of this brief review was to highlight the importance of some immune response genes and their correlation with the clinical forms of leprosy, as well as their implications for disease resistance and susceptibility.

## 1. Overview of Leprosy

Leprosy is a chronic infectious disease of slow evolution caused by *Mycobacterium leprae*, which primarily affects the skin and peripheral nerves and may manifest in different clinical forms. There is strong evidence for a genetic basis for host disease per se susceptibility to and its subtypes [1]. 

Currently, Brazil is the second in the world in absolute number of cases of leprosy [2]. Patients with leprosy can show a broad spectrum of clinical symptoms. The tuberculoid form (TT) of leprosy consists of well-defined lesions, few bacilli, and vigorous cell-mediated immunity (CMI). On the other hand, lepromatous leprosy (LL) presents as many skin lesions with uncontrolled proliferation of leprosy bacilli and inefficient CMI. Borderline leprosy manifests clinically and immunologically with characteristics between the poles of the spectrum of leprosy and may be classified as 3 subtypes: borderline lepromatous (BL), borderline borderline (BB), and borderline tuberculoid (BT) [3]. 

Most individuals develop sufficient immunity against *M. leprae* with no signs of clinical disease. However, in a small proportion of exposed individuals, leprosy can manifest in an array of clinical forms, ranging from the localized tuberculoid to the systemic lepromatous disease. Typically, Th1- and Th2-type immune responses are initiated against the pathogen [4]. Evidence suggests that the incidence of infection in the population is probably much higher than the incidence of clinical leprosy, because a small proportion (about 5%) of infected individuals develop clinical symptoms and the rest can develop subclinical infections or heal spontaneously. This may be due in part to environmental factors such as nutrition, genetic differences, or bacterial [5]. 

The clinical and pathological spectrum of leprosy can be explained by genetic differences in host resistance. While some loci affect intrinsic susceptibility to LD, others modify the clinical form of the disease [6]. This review may help to clarify the mechanisms immunopathogenics of *M. leprae*. Studies of immune response genes in patients with leprosy can be used as a research tool in assisting genetic characterization of leprosy patients, thus allowing the determination of a possible association between these gene combinations and the development of leprosy and its clinical forms.

Leprosy has long been considered a complex disease. In the past few years, several studies have attempted to characterize genes associated with leprosy, as well as their contribution to the development of the various clinical forms. Immune response genes have been associated with pathogenesis of different forms of leprosy. This review discusses the role of the human leukocyte antigen (*HLA*), Killer cell immunoglobulin-like receptors (*KIRs*), and MHC class I chain-related (*MIC*) genes, as well as polymorphisms of cytokines, in leprosy and their implications for resistance and susceptibility to the disease.

## 2. Strategy for Screening and Selecting Studies

This review about host genetic polymorphism studies, as well as the current status of genome-wide association studies and their influence on leprosy selected original articles carried out on humans that were found in the databases of PubMed (U.S. National Library of Medicine), LILACs (Latin American and Caribbean Center on Information in Health Sciences), and Google Scholar. The research period covered included the limit of databases until March 2013. There was no restriction regarding language. In the PubMed database MeSH (Medical Subject Heading) terms were used and in the LILACs descriptors were used. In order to retrieve articles of interest, free terms were used in the LILACS and Google Scholar. The MeSH terms, descriptors, and free terms were organized according to thematic groups: (i) HLA and Leprosy (“Leprosy” OR “Leprosy, Multibacillary” OR “Leprosy, Paucibacillary” OR “Leprosy, Tuberculoid” OR “Leprosy, Lepromatous” OR “Leprosy, Borderline” AND “HLA antigens/genetics”); (ii) KIR genes and Leprosy (“Leprosy” AND “Receptors, KIR”); (iii) MIC genes and Leprosy (“Leprosy” AND “MHC class I-related chain A”); (iv) Cytokine genes and Leprosy (“Leprosy” OR “Leprosy, Multibacillary” OR “Leprosy, Paucibacillary” OR “Leprosy, Tuberculoid” OR “Leprosy, Lepromatous” OR “Leprosy, Borderline” AND “Cytokines/genetics” OR “Receptors, Cytokine/genetics” OR “Chemokines/genetics”); (v) Genome-wide association study and Leprosy (“Leprosy” AND “Genome-Wide Association Studies”). The immune response genes, as *HLA*, *KIR*, *MIC,* and cytokines, and their association with leprosy were presented. 

Screening the PubMed, LILACs, and Google Scholar databases identified 326 potentially relevant citations. Of these, 260 citations were excluded after evaluating the title and the abstract, because they did not comply with the inclusion criterion, no human, aim, originality, duplicate articles and that could not be downloaded or accessed in full length from journal archives. 64 articles related to immune response genes in association with leprosy and 19 articles more which were added from reference list, adding 83 original articles on human infections included in this review were selected.

The main characteristics of the studies selected, the populations under study, the target genes, the number of individuals, and the main finding for each are shown in Tables 1, 2, and 3.

## 3. HLA and Leprosy

During infection caused by *M. leprae*, *HLA* alleles influence not only susceptibility and resistance to leprosy, but also the course of the disease. The main role of HLA molecules is to present peptides derived from *M. leprae *to T cells of the host [15]. An individual that has a particular combination of *HLA* alleles that are not linked to the peptide in an appropriate way, or for whom the HLA-peptide linkage does not elicit a proper lymphocyte response, will be more susceptible to infection than an individual that linked to the peptide in an appropriate way [18]. In patients whose HLA systems offer protection against the disease, these genes likely select and stimulate T cells to multiply and eliminate the agent via inflammatory cytokine production which destroy infected cells [15, 71]. Several studies have consistently reported the involvement of *HLA* alleles and haplotypes, mainly of class II genes, as important genetic factors controlling susceptibility to different forms of leprosy [71]. According to Ridley and Jopling (1966), the clinical manifestation of leprosy depends on the type of immune response that is initiated by the host and the balance between T-helper (Th)1 and Th2 responses may be partially controlled by the mechanism of antigen presentation involving HLA molecules [35, 71]. The tuberculoid (TT) form of leprosy is associated with a Th1 (cellular) immune response, characterised by the production of proinflammatory cytokines that can participate in the clearance of the bacillus. However, the lepromatous (LL) form of leprosy is associated with a Th2 (humoral) immune response, which is characterised by an immunosuppressive cytokine environment, making this type of response problematic for the host [3, 72].

## 4. Classical *HLA* Class I Genes

Several studies comparing *HLA* class I gene frequencies in leprosy cases and controls have found associations either with the polar forms of leprosy or with LD. Nevertheless, results have been inconsistent.

Earlier, association studies showed HLA-Aw21 as a factor of susceptibility to TT in Ethiopian patients [7], while HLA-A9 in India, HLA-A2 in Thailand and Korea as a factor of resistance to leprosy [8, 9, 13]. In leprosy patients from Iran, HLA-B35 antigen was increased, while HLA-A1 was decreased in LL patients [10]. The HLA-B40 antigen and HLA-A2-B40, HLA-A11-B40, and HLA-A24-B40 haplotypes were frequent among Indian leprosy patients [11], while HLA-A11 and HLA-A33 were increased among Korean LL patients [13].

In Indian patients, an increasing frequency of HLA-A11 [14] and HLA-B60 [15] antigens have been observed in LL patients. In southern Chinese, significantly decreased HLA-B46 was found in multibacillary leprosy [16]. In a Turkish LL case-control study, HLA class I serotypes A9, A10, Bw4, Bw6, Cw1, and Cw2 were significantly overrepresented, and serotypes A3 and B49 were significantly underrepresented in the LD patients [17].

Subsequently, with the advent of molecular genotyping, *HLA* class I alleles were determined in multibacillary leprosy patients, resulting in a positive association with HLA-A*02:06, A*11:02, B*18:01, B*51:10, C*04:07, and C*07:03 alleles, and a negative association with C*04:11 [18]. The A*11-B*40 haplotype was increased in multibacillary leprosy patients compared to controls [19].

Recent studies have shown a positive association between LD and HLA-A*11, HLA-B*38, and HLA-C*12, as well as a negative association with HLA-C*16. When groups were stratified, HLA-B*35 and HLA-C*04 were shown to be protective against lepromatous leprosy, while HLA-C*07 was shown to be a susceptibility variant [20]. Further, the allele HLA-C*15:05 was related to phenotype LD in certain populations from India and Vietnam [21].  Table 1 summarizes these findings. 

## 5. Classical *HLA* Class II Genes

According to some studies, the main restriction determinants for *M. leprae* reside on DR, and not DP or DQ molecules [73, 74]. The HLA-DR2 molecule [12, 13, 15, 21–24, 29, 32], later identified as DRB1*15 and DRB1*16 variants, is primarily associated with leprosy (LD or different clinical forms) in Indian, Japanese, Brazilian, and Chinese patients [25–27, 30, 33, 35, 36, 38, 39, 42].

In Indian patients, DRB1*15:02 was associated with TT [24, 25], whereas DRB1*15:01 was associated with LL [25]. DRB1*15:01 and *15:02 alleles differ from each other by a single amino acid at codon 86. Class II molecules have polymorphic pockets that accommodate the side chains of bound peptides. The codon 86 residue lies in binding pocket 1. In another Indian study, both DRB1*15:01 and *15:02 were found to be associated with tuberculoid leprosy, [27] indicating that the residue in pocket 1 may not be involved in determining the outcome of leprosy infection. Instead, it appears that certain residues that contribute to the net charge in the putative peptide-specific binding pocket 4 may be more important [75]. It is hypothesized that net negative or neutral charges in binding pocket 4 cause poor binding of the DRB1 molecule to *M. leprae* antigens. HLA molecules with the highest affinity to peptide produce the greatest T-cell proliferation and IFN-*γ* response [76], and the peptide presentation by low affinity class II molecules may result in muted cell-mediated immunity [75]. Alternatively, peptide presentation by specific class II molecules may result in activation of suppressor/regulatory T-cells [77].

Studies involving HLA-DRB1 have found a link between innate and T-cell-mediated immunity [78, 79], and results obtained from a multiple sclerosis study show that the presence of a VDRE (vitamin D response elements) in the proximal promoter region of the *HLA-DRB1* gene increased gene expression and imparts 1,25-(OH)2-D3 (Vitamin D) sensitivity to the DRB1*15:01 allele [79].

These observations point to the need to apply this possibility of association between these genetic variants and leprosy pathogenesis, since vitamin D, itself, may have a direct effect on leprosy through its receptors, VDR, or may influence leprosy through indirect effects [79].

Amino acid residues involved in the peptide binding groove of *HLA-DRB1* alleles were examined in three Nigerian ethnic groups (Bini/Igbo, Yoruba, and Efik) with leprosy. Nine positively charged motifs and 2 others with neutral charge in the peptide binding groove were detected. These motifs were more frequent in leprosy patients than was expected by chance. In contrast, 5 motifs with negative or “modified” neutral charges in the pocket were negatively associated with leprosy. Therefore, the clinical outcome of infection by *M. leprae* is largely determined by a shared epitope in *DRB1* alleles characterized by several motifs [75].

In leprosy patients from a Javanese population in Yogyakarta, Indonesia, HLA-DRB1*02 was associated with susceptibility to LL, while HLA-DRB1*12 was associated with resistance [28]. Risk for leprosy associated with the DRB1*10 allele has been described in Turkish, Vietnamese, and Brazilian populations [17, 36], whereas the HLA-DRB1*14 allele was associated with the TT group in a population from north-eastern Brazil [42] and DRB1*14:01 and DRB1*14:06 were associated with leprosy per se in Argentinean population [37]. A protective effect on leprosy has been described for DRB1*04 in Brazilian, Korean, Japanese, Vietnamese, Argentinean, and Taiwanese populations [13, 30, 34, 36, 40]. Associations between HLA class II and leprosy are summarized in Table 2.

The HLA complex has been studied in leprosy patients due to the direct involvement of these alleles in the immune response. In terms of both infection control and the manifestation of the different clinical forms, investigation of *HLA* genes may elucidate mechanisms of susceptibility and resistance, as well as disease course.

Even though genetic epidemiology data in leprosy involving alleles *HLA* is extensive, results should be cautiously interpreted due to the strong linkage disequilibrium across the alleles in this region, the common occurrence of weak study designs, and publication bias of positive results. Furthermore, functional data to support these associations are required.

## 6. *KIR *Genes and Leprosy

Killer cell immunoglobulin-like receptors (KIRs) are members of a group of regulatory molecules found on natural killer (NK) cells. These proteins are encoded by a complex of genes located in the Leukocyte Receptor Complex on chromosome 19p13.4, which has many polymorphisms that may be related to resistance to infection [80]. Known roles of NK cells include modulation on the immune system by the production of cytokines, as well as direct elimination of infected cells [81]. KIR molecules are either activating or inhibitory to NK cells. Inhibitory molecules (KIR2DL1, 2DL2, 2DL3, 2DL4, 2DL5, 3DL1, 3DL2, and 3DL3) function via the well-documented immunoreceptor tyrosine-based inhibitory motifs (ITIMs) [81]. The phosphorylated ITIMs serve as efficient recruitment points for the cytosolic protein tyrosine phosphatases, SHP-1 and SHP-2, resulting in the dephosphorylation of substrates critical for cellular activation [81].

Activating receptors (2DS1, 2DS2, 2DS3, 2DS4, 2DS5, and 3DS1) have truncated cytoplasmic domains lacking ITIMs but possess a charged residue (ITAMs) in their transmembrane domains that mediates interaction with the DAP-12 signal transduction chain. DAP-12 is a member of the immunoglobulin super family encoded at the centromeric end of the LRC. DAP-12 activation then leads to enhanced degranulation and production of cytokines and chemokines [82]. Studies performed over the last few years have revealed extensive diversity at the *KIR* gene locus, stemming from both its polygenic and multiallelic polymorphisms [83, 84].

Biologically, NK cell reactivity against target cells is partially based on the presence of KIRs and their cognate ligands, the HLA class I molecules. Some combinations of HLAs and KIRs may result in activation or inhibition of NK cells. It seems likely that NK receptor variants may be risk factors for infectious diseases in addition to HLA variants, as has been reported in leprosy. To further elucidate the balance between inhibitory and activating KIRs in the context of disease pathogenesis, continued epidemiological analysis of KIRs and disease should be pursued [85].

There are many studies showing the influence of *KIR* genes and their ligand pairs on the role of various infectious diseases. However, to the best of our knowledge only one study has explored the role of *KIR* genes in the pathogenesis of leprosy [82].

According to Franceschi et al. [82], a significant difference between *KIR *genes in TT and LL patients has been observed. In TT patients, the frequency of *KIR2DS3* (38.1%) was significantly higher than in LL patients (18.5%), and the frequency of *KIR2DS2* showed a trend of being higher in TT patients (61.9%) compared to LL patients (43.1%). *KIR2DS3* and *KIR2DS2 *are activator genes in linkage disequilibrium. Tuberculoid patients with both activator genes could develop a better NK-cell activation and then a more efficient cell-mediated immune response, with a milder manifestation of the disease. When KIR inhibitor genes and their HLA ligands were analyzed, TT patients had low frequencies of these KIRs in association with their correlated ligands, conferring a reduced NK cell inhibition and resulting in a protective mechanism against the most severe forms of the disease.

In the same study, patients with the form BB were observed to have a higher frequency of *KIR3DL2*-*A3/11* genes (40.0%) compared to the control group (24.6%) and LL patients (20.0%). In contrast, a reduced frequency of *KIR2DL1* with the *C2* as a ligand was found compared to TT patients (48.9% versus 76.3%) and the control group (48.9 versus 66.4%). This balance between these interactions may explain the undefined characteristics observed in BB patients.

According to Parham [86], of the family of KIR2DL molecules, KIR2DL1 with the ligand C2 is the most potent inhibitor. In the study by Franceschi et al. [82], an increased frequency of homozygous *C2*/*C2* was observed in TT patients compared to BB patients and to control group, suggesting that TT patients may be more susceptible to infection than the control group.

## 7. *MICA *Genes

In 1994, Bahram et al. [87] and Leelayuwat et al. [88] independently identified a new set of *loci* called MHC class I chain-related genes (*MIC*). The *MIC *family has two members*, MICA *and *MICB*, and 5 pseudogene members: *MICC*, *MICD*, *MICE*, *MICF, *and *MICG*. *MICA *is located at the centromeric end of the classical *HLA *class I region, approximately, 46.4 kb from *HLA-B* [89]. *MIC *genes encode a cell-surface glycoprotein of 383 amino acids, which is expressed in keratinocytes, fibroblasts and gastrointestinal epithelium, and several other cell types [90]. Exons 2, 3, and 4 of the gene encode three extracellular domains (*α*1, *α*2, and *α*3, resp.), while exon 5 encodes the transmembrane domain. Amino acid sequence alignments with classical HLA class I chains reveal between 15 and 21% homology in *α*1 and *α*2 domains but between 32% and 36% homology in the *α*3 domain [87].

Studies have shown that MICA works as a ligand for NK cells, *γδ* T cells, and *αβ* CD8+ T cells, which express a common activating NK cell receptor NKG2D [91]. NKG2D recognizes the human MICA protein in conjunction with a transmembrane signaling adaptor protein, DNAX-activation protein (DAP) [92].

Within exon 5, there is a short tandem repeat (STR) of GCT triplets in varying lengths [93]. This STR is commonly referred to as an “A” followed by the number of GCT repeats and occasionally a “1”, which reflects the presence of a G insertion (e.g., *A4*, *A5*, and *A5.1*). This information about exon 5 in *MICA *may therefore be of importance as polymorphisms in the transmembrane domain were correlated with the induction of autoreactive CD8+ cytotoxic T lymphocytes [94]. In addition, a G insertion within the exon 5 STR leads to a premature stop codon, which is translated into a truncated protein with impaired function [95].

Similar to classical *HLA*, *MICA *displays a high degree of allelic polymorphism within the nonclassical *HLA *gene *loci*, which results in MICA polymorphic residues that are positioned on the outer edge of an antigen-binding cleft, unlike MHC class I molecules [96], and they may have a role in the innate immune response to infection.

Since MIC expression is inducible by heat, viral infection, inflammation, and DNA damage, the molecules may be markers of stress in cells.

## 8. *MICA *and Leprosy

Studies linking *MIC *genes and leprosy are limited. Wang et al. [16] analyzed 69 southern Chinese leprosy patients and observed that *MICA-A5* allele showed a tendency to be negatively associated to multibacillar leprosy but not to paucibacillar. In the same group of patients, a negative association between the *HLA-B46*/*MICA-A5* haplotype and leprosy was found, suggesting that the *HLA-B46*/*MICA-A5* haplotype is significantly associated with resistance to leprosy. On the other hand, Tosh et al. [33] provided strong evidence that truncated MICA protein encoded by the *MICA-A5.1* allele plays a role in leprosy susceptibility in South Indian families.

In the study performed in southern Brazil by Sacramento et al. [96], 223 patients with leprosy from towns in the northern and northwestern regions of the State of Paraná participated. MICA*002, *008, *004, and *009 alleles were the most frequent, totalling 74.0% of all alleles in leprosy patients and 68.5% in the control group. There was only one significant difference: the frequency of the MICA*027 allele was higher in the control group compared to patients with LD. The alleles MICA*010 and MICA*027 had significant differences in multibacillary (LL, BB, and BL) patients compared to the control group. For the paucibacillary (TT and BT) group, no difference was found.

In this context, the MICA*027 allele was associated with protection against leprosy *per se *and the multibacillary subtypes. Individuals with the MICA*027 variant have normal expression of A5, a transmembrane domain which enables the interaction between MICA and NKG2D, activating NK cells.

Finally, these results suggest the influence of *MICA *alleles in the development of the leprosy and their clinical forms and need to be replicated.

## 9. Cytokines

An important factor that directs the clinical course of leprosy is the presence of proinflammatory and anti-inflammatory cytokines. Paucibacillary patients show a pattern of CMI of the Th1 type, which is characterized by the production of IFN-*γ*, IL-2, IL-7, IL-12, IL-15, and IL-18 in skin lesions. Conversely, multibacillary patients present a Th2 response with production of TGF-*β*1, IL-4, IL-5, and IL-10 in skin lesions with high antibody production, but insufficient CMI [97, 98].

SNPs are the most abundant source of genetic variation in the human genome, which can lead to differences in expression of proteins, causing structural and functional changes. Linking SNPs with the phenotypes of human diseases has great potential for direct clinical application, providing more accurate genetic markers for diagnosis and prognosis, and possibly new therapeutic targets [99]. Some SNPs in cytokine genes have been described as important genetic factors in the occurrence of different clinical forms of leprosy.

## 10. Cytokines and Leprosy

The gene encoding tumor necrosis factor (*TNF*) is located in the MHC region on chromosome 6. This cytokine exists in soluble and transmembrane forms [100] and is produced by cells of the immune system, tumor cells, and other cell types in response to inflammatory stimuli, infection, or stress [100]. There is ample evidence of the involvement of cytokines, especially tumor necrosis factor-alpha (TNF-*α*), in the immune response to leprosy. They may have a beneficial role in host defense but, if produced at high levels, cause tissue damage [101].

Studies conducted in Brazil by Santos et al. [43], Moraes et al. [44], Santos et al. [45], Franceschi et al. [46], and Cardoso et al. [47] indicated the association of *TNF-308A* (rs1800629) allele with a protective effect against the development of the disease. Vanderborght et al. [101], in a study in Rio de Janeiro, observed that patients possessing an A allele in the promoter region of *TNF-308* had a lower bacteriological index (BI), whereas the carriers of the A allele in the promoter region of *TNF-238* (rs361525) had higher BI.

A study in Nepal in 2010 by Sapkota et al. [48] showed results similar to Brazilian studies in relation to *TNF-308A* allele. However, studies conducted in an Indian and Thai populations [49, 50] showed a higher frequency of *TNF2* allele (with substitution G>A at position 308 in the *TNF *promoter region) in lepromatous and multibacillary patients, respectively, compared to the control group, indicating that this allele is associated with susceptibility to this form of disease. Nevertheless, a linkage study conducted in six French Polynesian families for Levée et al. [51] found no evidence of linkage between the loci *G1M*, *G2M*, *KM*, *IL-1* beta, *TNF-alpha* (1, 2), and *TNF-alpha* (A, G) and leprosy.

In the study multicase leprosy families from north-eastern of Brazil, the combined segregation and linkage analysis to the major locus showed strong linkage to *HLA* class II and tumour necrosis factor genes. Extended transmission disequilibrium testing, using multiple affected family members, demonstrated that the common allele TNF*1 of the −308 promoter region polymorphism showed linkage and/or association with disease per se, at a high level of significance. Two locus transmission disequilibrium testing suggested susceptibility (TNF*1/LTA*2) and protective (TNF*2/LTA*2) haplotypes in the class III region. Taken together the segregation and HLA analyses suggest the possibility of more than one susceptibility locus to leprosy in the MHC [31].

In a recent study in Mexico [52], no association was found between *TNF-308G/A *and leprosy, suggesting that other polymorphisms may be important in susceptibility to leprosy in this population. However, a study performed in a population from Northern India [53] provided further evidence for the role of variants *BAT1-LTA-TNF-BTNL2 *genes in susceptibility to leprosy. According to authors, the combination of low T-cell inhibition status of BTNL2, less inhibition of TNF by BAT1, and low TNF expression may provide protection from leprosy, which may be stronger in the presence of high TNF producer allele genetic background. 

Interleukin 10 (IL-10) is a cytokine produced by monocytes and activated T cells. It is deeply involved in the regulation of inflammatory and immunological reactions. Its effects do not only affect the immune system but can influence many physiological processes, including angiogenesis, tumorigenesis, and infection. Several polymorphisms have been observed in the *IL10* gene, including 6–11 CA repeats-microsatellite polymorphisms, and three point mutations: −1082 (G/A) (rs1800896), −819 (C/T) (rs1800871), and −592 (C/A) (rs1800872) [102]. 

Recently, in Mexican patients, Velarde-Félix et al. [52] found no statistically significant difference in the frequency of *IL10*−*819C* allele in patients and controls. However, in a Brazilian population, Pereira et al. [54] had reported that the *IL10*−*819T* allele was associated with leprosy in both a case-control study and in a meta-analysis.

Similar results were found in another Brazilian population of Rio de Janeiro by Santos et al. [45], where the *IL10*−*819TT* genotype was significantly higher in patients than in healthy controls, and the frequency of the *IL10*−*819T* SNP was greater in paucibacillary patients compared to multibacillary or among control subjects. However, in Colombian patients, the genotypes C/C and C/T in the SNP −819 and C/C and C/A in the −592 SNP were positively associated to leprosy. The haplotypes −819C−592C and −1082A−819C−592C showed significant association and these same haplotypes in homozygosis conditions were also associated with leprosy [55].

In another study, Moraes et al. [56] observed that in patients from the same Brazilian region the haplotype *IL10*−*3575A/*−*2849G/*−*2763C *was associated with resistance to leprosy and development of more severe forms of the disease, and that the haplotype *IL10*−*3575T*/−*2849A*/*2763C* was associated with susceptibility to LD.

In a study conducted in India, Malhotra et al. [57] observed that the extended haplotype *IL10*−*3575T/*−*2849G/*−*2763C/*−*1082A/*−*819C/*−*592C* conferred resistance to leprosy *per se* and to development of more severe forms of disease, whereas the haplotype *IL10*−*3575T/*−*2849G/*−*2763C/*−*1082A/*−*819T/*−*592A* was associated with the risk of developing a more severe form of the disease. A study in a population of southern Brazil by Franceschi et al. [46] showed a lower frequency of haplotype *IL10*−*1082G/*−*819C/*−*592C* in patients with the lepromatous form of the disease compared to the control group. The results of these studies strongly suggest the involvement of SNPs in the promoter region of the *IL10* gene in leprosy.

IL-12 consists of two covalently linked subunits: p35 and p40. Antigen-presenting cells, specifically dendritic cells and macrophages, are the main producers of this cytokine. The effects of IL-12 are mainly controlled by the level of transcription of p40 and expression of IL-12R. IL-12 is produced quickly after infection and acts as a proinflammatory cytokine by inducing IFN-*γ* production and enhancing the proliferation and cytotoxicity of NK and T cells [103].

According to Morahan et al. [58], in Indian patients, subjects with leprosy were less likely to have the 3′UTR genotype associated with lower IL-12B expression. However, in Korean patients, Lee et al. [59] found no significant differences in allele frequencies of *IL12RB1* between leprosy patients and the control group [59]. Now, in relation to gene in the 5′ flanking region of *IL12RB2*, Ohyama et al. [60] determined the functional effects of these SNPs on NK-cell activity, including IFN-*γ* production and *IL-12RB2* gene expression. The results suggest that these SNPs in *IL12RB2* have differential effects on cellular activation of T and NK cells [60].

In Western Mexico, Alvarado-Navarro et al. [61] found that the 1188A/C polymorphism in the 3′UTR of *IL12p40* gene was associated with greater susceptibility to lepromatous leprosy, independent of the expression levels of IL-12 p40. Conversely, Jesús Salvador et al. [62] in a study with Mexican patients found no significant association between genotype and allele frequencies of the 1188A/C polymorphism and lepromatous leprosy [62].

Recently, Liu et al. [63] conducted a multiple-stage genetic association study in leprosy patients from China and discovered associations implicating *IL18RAP/IL18R1* (rs2058660) and* IL12B* (rs6871626) as susceptibility genes for leprosy. 

The *IFNG* gene encodes the IFN-*γ* cytokine, which plays a key role in host defense against intracellular pathogens. SNPs in *IFNG* were evaluated in several epidemiological studies; the SNP *INFG+874T/A* (rs2430561), more specifically, the allele *INFG+874T* has been associated with protection against infectious diseases [104].

In patients from São Paulo and Rio de Janeiro, two independent studies conducted by Cardoso et al. [64] showed that the *INFG+874T* allele conferred protection against leprosy. Recently, in Chinese patients, Wang et al. [65] found no association between *INFG+874T/A* and leprosy. However, the variant rs3138557 in the *IFNG *gene had many CA-repeat alleles and they observed that the alleles *INFG* (10CA), INFG (13CA), and *INFG* (15CA) had a higher frequency in patients, especially in multibacillary compared to the control group (3.2 versus 0.6%; 21.3 versus 18.6%; and 21.8 versus 18.0%, resp.), and that the allele *INFG* (17CA) was more frequent in paucibacillary patients than in controls (2.8 versus 1.2%). In patients from Amazonas state, Brazil, there were no significant differences between patients and control subjects, as well as according to Ridley-Jopling classification. However, the A/A genotype and the allele *INFG* (16CA) were significantly associated with paucibacillary compared to multibacillary patients [66]. 

In a population of Brazilian patients, Reynard et al. [67] observed that a higher frequency of alleles *INFG* (15CA),* INFG* (16CA), and *INFG *(17CA) was positively associated with leprosy, which indicates that the *IFNG* gene polymorphism may contribute to the course of infection.

In Korean patients, no significant differences were found in allele frequencies* IFNGR1* (interferon *γ* receptor 1) between leprosy patients and the control group [59]. However, a case report showed that the *IFNGR1* polymorphism at position −56T/C was positively associated with an increased susceptibility to leprosy, in Iranian children of the same family [68]. 

Polymorphisms in the *IL4* gene influence the production of IL-4, an important anti-inflammatory cytokine generated by T-helper type 2 (Th2) cells, which have multiple roles in the immune system. Three polymorphisms in *IL4* have been described: a single base polymorphism −590T/C (rs2243250) in the promoter region, polymorphism +33C/T (rs2070874) in exon 1, and type VNTR polymorphism (variable number of tandem repeat) in intron 3. In a Chinese study, Yang et al. [69] observed that the* IL4−590T/C *and C/C genotypes, and the −590C allele were less frequent in leprosy patients than in the control group (25 versus 29.9%; 3.9 versus 7.5%; and 16.4 versus 22.5%, resp.), suggesting that the allele *IL4*−*590C* is associated with resistance to leprosy in this population.

Interleukin-6 (IL-6) is a pleiotropic cytokine, produced by different cell types, such as macrophages, fibroblasts, and endothelial cells. IL-6 plays an important role in a wide range of processes, such as immune response, acute phase reactions, and hematopoiesis [105].

Recently, in a case-control study, Sousa et al. [106] observed a correlation between plasma levels of IL-6 and *IL6* genotypes in patients with Type-2 reactions in leprosy. Type-1 and Type-2 leprosy reactions are aggressive inflammatory episodes with highly variable incidence rates across populations but affect up to 50% of leprosy patients. Identification of genetic factors predictive of leprosy reactions could have a great impact on prevention strategies.

A study conducted in MassARRAY platform, carried out by Aggarwal et al. [70], in the Indian population investigated the association of 51 SNPs in anti-inflammatory cytokine and receptor genes with susceptibility to leprosy. Significant associations with leprosy were observed for 8 polymorphisms (rs1800871, rs1800872, and rs1554286 of *IL10*, rs3171425 and rs7281762 of *IL10RB*, rs2228048 and rs744751 of *TGFBR2*, and rs1800797 of *IL6*). The study revealed a greater association of these polymorphisms with the risk for leprosy than those obtained for any SNP studied individually. This provides an interesting insight on the cumulative polygenic host component that regulates leprosy pathogenesis [70].  Table 3 summarizes these findings. 

Studies have been carried out in order to investigate a possible combined effect of *HLA *genes and cytokines genes in leprosy, more specifically *TNF* gene and *HLA* class II [31, 49]. However, the results are inconsistent. The first study by Roy et al. [49] did not find linkage disequilibrium between *TNF2* allele and *HLA* class II, showing that these genes appear to be independent, whereas Shaw et al. [31] showed strong linkage between *HLA* class II (*HLA-DQB1*, *P* = 0.000002; *HLA-DQA1*, *P* = 0.000002; *HLA-DRB1*, *P* = 0.0000003) and *TNF* genes (*TNF*, *P* = 0.00002; *LTA*, *P* = 0.003). More studies are needed to clarify this linkage because polymorphisms within the TNF gene, which is located close to the class II region, may lead to variability in TNF-*α* secretion during the leprosy infection [49]. This is significant, since in mycobacterial infections, TNF-*α* promotes host defense mechanisms and granuloma formation, but high concentrations of TNF-*α* are associated with immunopathology [49]. 

## 11. Genome-Wide and Leprosy

Finally, we will summarize findings from some important genome-wide association studies of leprosy. The first GWAS of leprosy susceptibility reported convincing associations with markers in six genetic loci: *HLA-DR-DQ* (rs602875, *P* = 5.4 × 10^−27^, OR = 0.67), receptor-interacting serine-threonine kinase 2 (*RIPK2*) (rs42490, *P* = 1.4 × 10^−16^, OR = 0.76), tumor necrosis factor [ligand] superfamily member 15 (*TNFSF15*) (rs6478108, *P* = 3.4 × 10^−21^, OR = 1.37), laccase (multicopper oxidoreductase) domain-containing 1 (*LACC1*; previously known as *C13*orf31) (rs3764147, *P* = 3.7 × 10^−54^, OR = 1.68), coiled-coil domain-containing 122 (*CCDC122*) (rs3088362, *P* = 1.4 × 10^−31^,  OR = 1.52), and nucleotide-binding oligomerization domain-containing 2 (*NOD2*) (rs9302752, *P* = 3.8 × 10^−40^, OR = 1.59) [107].

Subsequently, associations between leprosy and the *HLA-DR-DQ* region, *LACC1*, *CCDC122*, and the I602S functional SNP in the Toll-like receptor 1 (*TLR1*) gene were replicated in an Indian population [108, 109] and between the *HLA-DR-DQ*, *RIPK2*, *CCDC122*,* LACC1*, and* NOD2* in Vietnam [110]. 

Interesting, an association between *LACC1* (previous C13orf31) and *CCDC122* and susceptibility to Crohn's disease was related [111]. However, both genes were of unknown function and should be investigated in relation to their biologic function, which will probably clear a pathogenic mechanism of both diseases.

Recently, Yang et al. [112] carried out a genome-wide single nucleotide polymorphism (SNP) based linkage analysis using 23 pedigrees, each with 3 to 7 family members affected by leprosy, in China [112]. They suggested genomewide significant evidence for linkage on chromosome 2p14, and a suggestive evidence for linkage on chr.4q22 (rs1349350), chr.8q24 (rs1618523), and chr.16q24 (rs276990), as well as a moderate evidence for a linkage locus on chromosome 6q24–26 (rs6570858), overlapping a previously reported linkage region on chromosome 6q25-26 [112].

## 12. Conclusion

The analysis of genetic variants in the susceptibility to infectious diseases has been a topic widely discussed. Through various studies, it is known that the environment and the virulence of the pathogen are not sufficient to explain the different immune response patterns presented in the same population against a particular pathogen. The hypothesis of the existence of a complex network of factors acting simultaneously in infectious disease is recognized, and within this context, in leprosy the host immune response is a critical factor for the onset of the disease, and the levels of this response are influenced by the interaction of different genes. 

*M. leprae* can cause very different disease phenotypes in humans, probably due to individual variation in genetic profile and, consequently, in immune responses. Of the many reports of genes associated with leprosy, relatively few have been replicated in additional study populations. Further studies, involving a large number of genetic factors in populations from different parts of Brazil and the world, should be conducted to elucidate the interactions between these factors, which may be useful in the prognosis and clinical evolution of leprosy patients. 

The purpose of this brief review was to highlight the importance of some immune response genes and their correlation with the development of clinical forms of leprosy, as well as their implications for disease resistance and susceptibility.

## Figures and Tables

**Table 1:** Associations between HLA class I and leprosy.

Population	Study design	Sample size	Phenotype	Serotype, allele, or haplotype	Type of association	*P* or *Pc *	Ref.
Ethiopian	Case-control	20TT, 19LL, 36 controls	TT	Aw21	Susceptibility	*Pc* = 0.042	[7]

Indian	Case-control	30BT or TT, 40 controls	TT	A9	Resistance	*Pc* = 0.005	[8]

Thai	Case-control	26TT, 183 controls	TT	A2 Bw17	Resistance Susceptibility	0.01 < *P* < 0.05 0.01 < *P* < 0.05	[9]
	Case-control	70LL, 183 controls	LL	B7	Susceptibility	0.01 < *P* < 0.05	

Iran	Case-control	88LD, 125 controls	LL	A1 B35	Resistance Susceptibility	*P* < 0.05	[10]

Mumbai/Indian	Case-control	158LL, 150TT, 170 controls	LL LL LL TT/LL TT/LL	B-40 Aw19 A2-B40 A11-B40 A24-B40	Susceptibility Resistance Susceptibility Susceptibility Susceptibility	*Pc* = 0.0027 *Pc* = 0.02 *P* < 0.00025 *P* < 0.00025 *P* < 0.00025	[11]

Japanese	Case-control	295LL, 74TL, 110 controls	LL	B7 Bw54	Susceptibility Resistance	*Pc* = 0.044 *Pc* = 0.016	[12]

Korean	Case-control	157LD, 162 controls	LD	A2 A11 Aw33 Cw5	Resistance Susceptibility Susceptibility Resistance	*P* = 0.03 *P* = 0.03 *P* = 0.003 *P* = 0.001	[13]

Indian	Case-control	65ENL, 71LL	ENL	A11	Susceptibility	*Pc* = 0.0035	[14]

Indian	Case-control	68LL, 237 controls	LL	B60	Susceptibility	*Pc* = 0.00019	
	Case-control	138LD, 237 controls	LD	B60	Susceptibility	*Pc* = 0.031	[15]
	Case-control	20BB, 237 controls	BB	B40	Susceptibility	*Pc* = 0.018	

Southern Chinese	Case-control	50LL, 69 controls	LL	B46	Resistance	*P* < 0.01	[16]

Turkish	Case-control	80LD, 120 controls	LD	A9 A10 Bw4 Bw6 Cw1 Cw2 A3 B49	Susceptibility Susceptibility Susceptibility Susceptibility Susceptibility Susceptibility Resistance Resistance	*Pc* = 0.0004 *Pc* = 0.0226 *Pc* = 0.00003 *Pc* = 0.00001 *Pc* = 0.0080 *Pc* = 0.0055 *Pc* = 0.0040 *Pc* = 0.0035	[17]

Southern Indian	Case-control	32LD, 67 controls	LD	A*02:06 A*11:02 B*51 : 10 B*18:01 C*04:07 C*07:03 C*04:11	Susceptibility Susceptibility Susceptibility Susceptibility Susceptibility Susceptibility Resistance	*Pc* = 0.000007 *Pc* = 0.00001 *Pc* = 0.0000005 *Pc* = 0.007 *Pc* = 1.0 × 10^−9^ *Pc* = 0.000001 *Pc* = 0.001	[18]

Mumbai/Indian	Case-control	103LD, 101 controls	LD	A02 A11 A28 B12 B15 B40 Cw7 Cw3	Susceptibility Susceptibility Resistance Resistance Resistance Susceptibility Susceptibility Resistance	*Pc* = 0.0015 *Pc* = 0.009 *Pc* = 0.0014 *Pc* = 0.001 *Pc* = 0.05 *Pc* = 7.34 × 10^−7^ *Pc* = 2.26 × 10^−5^ *Pc* = 0.0002	[19]
	Case-control	32ML, 67 controls	ML	A*02:06 A*11:02 B*18:01 B*51:10 C*04:07 C*04:11 C*07:03	Susceptibility Susceptibility Susceptibility Susceptibility Susceptibility Resistance Susceptibility	*Pc* = 7.15 × 10^−5^ *Pc* = 0.00001 *Pc* = 0.007 *Pc* = 5.29 × 10^−6^ *Pc* = 5.12 × 10^−9^ *Pc* = 0.001 *Pc* = 1.97 × 10^−5^	
	Case-control	32ML, 67 controls	ML	A*11-B*40	Susceptibility	*Pc* = 0.002	

Brazilian	Case-control	224LD, 446 controls	LD	A*11 B*38 C*12 C*16	Susceptibility Susceptibility Susceptibility Resistance	*P* = 0.0345 *P* = 0.0402 *P* = 0.01 *P* = 0.0124	[20]
	Case-control	88LL, 48TT	LL	C*07 B*35 C*04	Susceptibility Resistance Resistance	*P* = 0.0211 *P* = 0.0156 *P* = 0.0464	

Vietnamese	family study	198 families	LD	C*15:05	Susceptibility	*P * = 0.0063	
	family study	292 families	LD	C*15:05	Susceptibility	*P* = 8.8 × 10^−5^	[21]

Indian	Case-control	364LD, 371 controls	LD	C*15:05	Susceptibility	*P* = 3.0 × 10^−8^	

MB: multibacillary leprosy; LL: lepromatous leprosy; BB: borderline borderline; TT: tuberculoid leprosy; ENL: erythema nodosum leprosum; LD: leprosy disease; ns: not significant; *Pc*: corrected *P* Value; Ref.: reference.

**Table 2:** Associations between *HLA* class II and leprosy.

Population	Study design	Sample size	Phenotype	Serotype, allele, or haplotype	Type of association	*P* or *Pc *	Ref.
Japanese	Case-control	295LL, 74TL, 110 controls	LL/TT LL	DR2 DRw9	Susceptibility Resistance	*Pc* < 0.008 *Pc* < 0.0001	[12]

Thai	Case-control	32TT, 32 controls	TT	DR2 DQw1	Susceptibility Susceptibility	*Pc* = 0.02 *Pc* = 0.008	[22]

Korean		157LD, 162 controls	LD	DR1 DR2 DR9 DR4 DRw53 DQw1 DQw3	Susceptibility Susceptibility Susceptibility Resistance Resistance Susceptibility Resistance	*P* = 0.02 *P* < 0.0001 *P* = 0.02 *P* < 0.0001 *P* < 0.0001 *P* < 0.0001 *P* < 0.0001	[13]

Turkish	Case-control	23LL, 27BL, 50 controls	LL/BL	DR2	Susceptibility	*P* = 0.015	[23]

Asian Indian	Case-control	23TT, 16PTB, 19 controls	TT	DRB1*15:02	Susceptibility	*P* < 0.05	[24]

Indian	Case-control	138LD, 237 controls	LD	DR2 DQw1 DQw7	Susceptibility Susceptibility Susceptibility	*Pc* = 0.00031 *Pc* = 0.0004 *Pc* = 0.00031	[15]
	Case-control	68LL, 237 controls	LL	DR2 DQw1	Susceptibility Susceptibility	*Pc* = 0.0063 *Pc* = 0.02	
	Case-control	30BL, 237 controls	BL	DR9 DQw7	Susceptibility Susceptibility	*Pc* = 0.04 *Pc* = 0.0006	

Indian	Case-control	28TT, 65LL, 47 controls	LL	DRB1*15 DRB1*15:01 DRB1*07:01 DRB5*01:01 DQB1*06:01 DQA1*01:02 DQA1*01:03 DQA1*02:01	Susceptibility Susceptibility Susceptibility Susceptibility Susceptibility Susceptibility Susceptibility Susceptibility	*P* < 0.0001 *P* < 0.001 *P* < 0.01 *P* < 0.001 *P* < 0.0001 *P* < 0.01 *P* < 0.01 *P* < 0.01	[25]
	Case-control	28TT, 65LL, 47 controls	TT	DRB1*15 DRB1*15:02 DQB1*0601 DQB1*05:03	Susceptibility Susceptibility Susceptibility Resistance	*P* < 0.01 *P* < 0.05 *P* < 0.01 *P* < 0.01	

Indian	Case-control	39TT, 20PTB, 46 controls	TT	DRB1*15:02 Haplotype: DRB1*1501-DRB5*0101-DQA1*0102-DQB1*0502	Susceptibility Resistance	*P* < 0.05 *P* < 0.05	[26]

Indian	Case-control	54TT, 44 controls	TT	DRB1*15	Susceptibility	*Pc* = 0.0063	[27]

Japanese	Case-control	38LL/BL, 79LD, 50 controls	BL/LL LD	DRB1*02 DRB1*12	Susceptibility Resistance	*P* = 0.037 *P* = 0.013	[28]

Brazilian	Case-control	32TT, 147 controls	TT	DR2	Susceptibility	*Pc* = 0.0132	[29]

Japanese	Case-control	93LD, 114 controls	LD	DRB1*04:05 DQA1*03 DQB1*04:01	Resistance Resistance Resistance	*Pc* < 0.05 *Pc* < 0.05 *Pc* < 0.05	[30]

Brazilian	TDT	73 families (147 sib-pairs)	LD	DQB1*02:01 DQB1*05:01 DRB1*7	Resistance Susceptibility Resistance	*Pc* = 0.008 *Pc* = 0.008 *Pc* = 0.036	[31]
	TDT	73 families (147 sib-pairs)	TT	DQB1*02:01 DQB1*05:01	Resistance Susceptibility	*Pc* = 0.024 *Pc* = 0.024	

Egyptian	Case-control	24LD, 30 controls	LD	DR2 DQ1	Susceptibility Susceptibility	*P* = 0.032 *P* = 0.015	[32]

Southern Indian	TDT	223 ASP	TT	DRB1*15(2) DRB1*09	Susceptibility Resistance	*P* = 0.012 *P* = 0.004	[33]

Argentinean	Case-control	70LL, 112 controls	LL	DQB1*02:01 DQB1*02:02 DQB1*02:03	Resistance	*Pc* = 0.02	[34]
	Case-control	19PB, 112 controls	TT	DRB1*04	Resistance	*Pc* = 0.0192	

North Indian	Case-control	34BT/TT, 79BL/LL, 111 controls	BL/LL	DRB1*15:01	Susceptibility	*P* < 0.05	[35]

Brazilian	Case-control	578LD, 691 controls	LD	DRB1*04 DRB1*07 DRB1*10 DRB1*12 DRB1*15	Resistance Resistance Susceptibility Resistance Susceptibility	*Pc* = 0.04076 *Pc* = 0.04753 *Pc* = 0.02102 *Pc* = 0.04399 *Pc* = 0.02288	[36]
Euro-Brazilian	Case-control	578LD, 691 controls	LD	DRB1*04/NN^c^ DRB1*07/NN^c^	Resistance Resistance	*Pc* = 0.01 *Pc* = 0.01	
Afro-Brazilian	Case-control	578LD, 691 controls	LD	DRB1*10/NN^c^ DRB1*15/NN^c^	Susceptibility Susceptibility	*Pc* = 0.024 *Pc* = 0.0002	
Vietnam	TDT	194 single-case families	LD	DRB1*10 DRB1*04	Susceptibility Resistance	*Pc* = 0.04 *Pc* = 0.03	

Argentinean	Case-control	71LD, 81 controls	LD	DRB1*14:01 DRB1*14:06 DRB1*08:08 DRB1*11:03	Susceptibility Susceptibility Resistance Resistance	*Pc* = 0.0011 *Pc* = 0.0011 *Pc* = 0.0006 *Pc* = 0.0004	[37]

Chinese	Case-control	305LD, 527 controls	LD	DRB1*15 DRB1*09	Susceptibility Resistance	*Pc* = 0.002 *Pc* = <0.001	[38]

Brazilian	Case-control	30BL, 178 controls	BL	DRB1*16:01	Susceptibility	*Pc* = 0.0208	[39]
	Case-control	63LL, 43TT	LL	DRB1*08	Susceptibility	*Pc* = 0.0481	

Taiwanese	Case-control	65LD 190 controls	ML	DRB1*04:05	Resistance	*Pc* = <0.0001	[40]

Brazilian	Case-control	17LL, 77 control	LL	DRB1*11	Resistance	*Pc* = 0.0132	[41]

Brazilian	Case-control	36LL, 85 control	LL	DRB1*16	Susceptibility	*Pc* = 0.0105	[42]
	Case-control	20TT, 85 control	TT	DRB1*14	Susceptibility	*Pc* = 0.032	

MB: multibacillary leprosy; PB: paucibacillary; LL: lepromatous leprosy; BL: borderline lepromatous; BB: borderline borderline; TT: tuberculoid leprosy; PTB: pulmonary tuberculosis; LD: leprosy disease; NN^c^: all not significantly different alleles collapsed into a unique group (i.e., DRB1*04, *07*, *10*, *12,* and *15*) [36]; ASP: affected sib-pair; ns: not significant; *Pc:* corrected *P* value; Ref.: reference.

**Table 3:** Associations between cytokine genes and leprosy.

Population	Study design	Sample size	Phenotype	Allele, genotype, or haplotype	Type of association	*P *	Ref.
Brazilian	Case-control	70LL, 85BL, 55BB, 2BT, 63TT, 10IL, 15 pure neural, 92 controls	BT/TT	*TNF-308A *	Resistance	*P* = 0.005	[43]

Brazilian	Case-control	74BT	BT/TT	*TNF-308A *	Resistance	ND	[44]

Brazilian	Case-control	210MB, 90PB, 92 controls 143MB, 79PB, 62 controls	LD PB LD	*TNF-308A* *IL10-819T* *IL10-819TT *	Resistance Susceptibility Susceptibility	*P* < 0.05 *P* < 0.01 *P* = 0.04	[45]

Brazilian	Case-control	43TT, 65LL, 50BB, 9IL, 240 controls	LD LL	*TNF-308A* *IL10-1082G/-819C/-592C *	Resistance Resistance	*P* = 0.02 *P* = 0.02	[46]

Brazilian	Family study case-control	363LD 1146LD, 1036 controls	LD	*TNF-308A *	Resistance Resistance	*P* < 0.04 *P* < 0.02	[47]

Nepal	Case-control	581(LL,BL,BB), 343(BT,TT); 101 controls	LD	*TNF-308A *	Resistance	*P* = 0.016	[48]

Indian	Case-control	121LL, 107TT, 160 controls	LL	*TNF-308A *	Susceptibility	*P* = 0.02	[49]

Thai	Case-control	24MB, 13PB, 140 controls	MB LD	*TNF-308A* *TNF-308GA *	Susceptibility Susceptibility	*P* = 0.04 *P* = 0.04	[50]

French Polynesian	Family study	6 families	LD	*IL-1 beta, TNF-alpha (1, 2),* and *TNF-alpha (A, G) *	ns		[51]

Brazilian	Multi case families study	76 families	LD	TNF*1 TNF*1/LTA*2 TNF*2/LTA*2	Susceptibility Susceptibility Resistance	*P* = 0.0001 *P* = 0.014 *P* = 0.001	[31]

Mexican	Case-control	62 cases, 144 controls	LL	*TNF-308G/A* *IL10-819C *	ns		[52]

Indian	Case-control	449PB, 473MB, 1670 controls	LD	*BAT1-LTA-TNF-BTNL2 *	Susceptibility	*P* < 0.0001	[53]

Brazilian	Case-control Meta-analysis	374 cases, 380 controls 2702 individuals (5 studies)	LD	*IL10-819T *	Susceptibility	*P* = 0.01	[54]

Colombian	Case-control	100 cases, 100 controls	LD	*IL10-819CC *and *CT* *IL10-592CC *and *CA* *IL10-819C-592C* *IL10-1082A-819C-592C *	Susceptibility Susceptibility Susceptibility Susceptibility	*P* < 0.001 *P* < 0.001 *P* < 0.001 *P* < 0.001	[55]

Brazilian	Case-control	131PB, 166MB, 283 controls	LD	*IL10-3575A/-2849G/-2763C* *IL10-3575T/-2849A/2763C *	Resistance Susceptibility	*P* = 0.005 *P* = 0.027	[56]

Indian	Case-control	144MB, 142PB, 266 controls	LD	*IL10-3575T/-2849G/-2763C/-1082A/-819C/-592C* *IL10-3575T/-2849G/-2763C/-1082A/-819T/-592A *	Resistance Susceptibility	*P* = 0.01 *P* = 0.0002	[57]

Indian	Case-control	80 cases, 89 controls	LD	*IL12B 3*′*UTR 2.2 *	Resistance	*P* = 0.001	[58]

Korean	Case-control	93LL, 94 controls	LL	*IL12RB1* *IFNGR1 *	ns		[59]

Japanese	Case-control	130LL, 46TL, 68 controls	LL	*IL12RB2-1035G* *IL12RB2-1023G* *IL12RB2-650delG* *IL12RB2-464G* *IL12RB2-1035A/-1023A/-650G/-464A *	Susceptibility	*P* < 0.001 *P* < 0.01 *P* < 0.001 *P* < 0.01 *P* < 0.039	[60]

Mexican	Case-control	44LL, 51 controls	LL	*IL12 3*′*UTR 1188A/C *	Susceptibility	*P* < 0.05	[61]

Mexican	Case-control	66LL, 140 controls	LL	*IL12 3*′*UTR 1188A/C *	ns		[62]

Chinese	Multiple-stage genetic association	4971 cases, 5503 controls	LD	*IL18RAP/IL18R1*(rs2058660)* IL12B*(rs6871626)	Susceptibility	*P* = 4.57 × 10^−19^ *P* = 3.95 × 10^−18^	[63]

Brazilian	Case-control	1045 cases, 1080 controls	LD	*IFNG+ 874T *	Resistance	*P* = 0.005	[64]

Chinese	Case-control	527 cases, 583 controls	LD LD, MB MB MB PB	*IFNG+ 874T/A* *IFNG(10CA),* *IFNG(13CA)* *IFNG(15CA)* *IFNG(17CA) *	ns Susceptibility Susceptibility Susceptibility Susceptibility	*P* = 0.001 *P* = 0.026 *P* = 0.007 *P* = 0.04	[65]

Brazilian	Case-control	108 cases, 113 controls	PB	*IFNG+874AA* *IFNG(16CA) *	Susceptibility Susceptibility	*P* = 0.028 *P* = 0.019	[66]

Brazilian	Case-control	10TT, 59BB, 27LL, 98 controls	LD	*IFNG(15CA), (16CA), *and* (17CA) *	Susceptibility	*P* = 0.01	[67]

Iranian	Case report	3 cases	LD	*IFNGR1-56T/C *	Susceptibility	ND	[68]

Chinese	Case-control	80PB, 352MB, 465 controls	LD	*IL4-590TC* *IL4-590CC* *IL4-590C *	Resistance Resistance Resistance	*P* = 0.044 *P* = 0.01 *P* = 0.001	[69]

Indian	Case-control	2447 cases, 1294 controls	LD	*IL-10 *(rs1800871, rs1800872, rs1554286); *IL-10RB *(rs3171425; rs7281762); *TGFBR2 *(rs2228048, rs744751); *IL-6* (rs1800797)	Susceptibility	*P* < 0.05	[70]

MB: multibacillary; PB: paucibacillary; LL: lepromatous leprosy; BL: borderline lepromatous; BT: borderline tuberculoid; TT: tuberculoid leprosy; LD: leprosy disease; ns: not significant; ND: no data; *P* value; Ref.: reference.
